# Cervical stump pregnancy 6 years after subtotal hysterectomy: a case report

**DOI:** 10.1186/s13256-019-2077-9

**Published:** 2019-05-08

**Authors:** Dawud Muhammed Ahmed, Abebe Assaye Fetene, Eyaya Misgan Asres, Amsalu Worku Mekonnen, Hailu Gashe Mamuye

**Affiliations:** 1 Bahir Dar University, Bahir dar, Ethiopia; 2 Funeteselam General Hospital, Finote Selam, Ethiopia

**Keywords:** Cervical stump, Ectopic pregnancy, Hysterectomy

## Abstract

**Background:**

Pregnancy following hysterectomy is very rare and may lead to significant morbidity, especially when diagnosis is delayed.

**Case presentation:**

A 32-year-old G3P3002 African woman presented with increasing abdominal distension and 1 day of worsening abdominal pain and vomiting. Her previous pregnancy had ended 6 years prior with a stillborn baby delivered by Cesarean hysterectomy after laboring at home for 1 day. At the time of current presentation, this patient was confused and irritable, with an undetectable blood pressure, tachycardia, labored breathing, and a distended and tender abdomen. Urine human chorionic gonadotropin was positive and a transabdominal ultrasound showed significant intraperitoneal fluid collections and a singleton, viable pregnancy consistent with 13 weeks of gestation. A diagnosis of hypovolemic shock secondary to ruptured ectopic pregnancy was made, and she underwent emergency laparotomy. Intraoperative findings included 4.5 liters of hemoperitoneum and a cervical stump pregnancy with active bleeding from a partially detached placental site.

**Conclusion:**

Any reproductive-aged woman with at least one ovary and a means for sperm to meet egg should be screened for pregnancy if she presents with an acute abdomen or abdominal or pelvic pain.

## Background

While a complete uterus is the typical site of gestation, it is not absolutely necessary for fertilization and implantation. Ectopic pregnancies are most commonly found in the fallopian tubes or on peritoneal surfaces in the pelvis, including on the ovaries and omentum [1]. Pregnancy after hysterectomy is extremely rare, with the first case of ectopic pregnancy after hysterectomy reported by Wendler in 1895 [2–4]. To the best of our knowledge, there are only 72 cases of post-hysterectomy ectopic pregnancy reported in the world literature [3].

## Case presentation

A 32-year-old G3P3002 African woman came from Yifag Kebele, Amhara Region, to Felege Hiwot referral hospital in Bahir Dar, Northwest Ethiopia in July 2016. She presented with abdominal pain and intractable vomiting of 1 day’s duration. She was also unable to pass feces and flatus and had developed progressive abdominal distension. She had a past medical history notable only for chronic gastritis for which she took unspecified medications and a past surgical history notable for a Cesarean hysterectomy after an intrauterine fetal demise during labor. As she had been told that her uterus was removed, she did not use contraception and had no menses. She was admitted to our surgical ward with a diagnosis of small bowel obstruction due to presumed post-operation adhesions and possible incisional hernia. She also had severe anemia and was resuscitated with 2 liters of normal saline and transfused with 2 units of blood. A plan was made to correct the hernia once she was stabilized. After 2 days in our hospital, however, her condition worsened and a consultation was made to Obstetrics and Gynecology for further evaluation.

On physical examination at the time of consultation, she was confused and irritable, with an undetectable blood pressure and a thready pulse of 132. She had labored breathing, pale conjunctiva, and a distended abdomen with a palpable mass below the midline surgical scar. An abdominal examination also revealed a fluid wave and hypoactive bowel sounds. Laboratory testing showed a white blood cell count of 12.9 × 10^3^ with 88.4% neutrophils and hemoglobin of 5.8 g/dl. Urine human chorionic gonadotropin (hCG) was positive. A transabdominal ultrasound showed a normal liver, spleen, pancreas, and kidneys. There was a significant debris-filled intraperitoneal fluid collection, especially on the right side of her abdomen, with a deepest pocket measuring 5 cm. No lymphadenopathy was seen. A singleton, viable pregnancy was identified measuring 13 weeks of gestational age with no gross abnormality and adequate amniotic fluid.

The Obstetrics and Gynecology team diagnosed hypovolemic shock secondary to ruptured ectopic pregnancy, and our patient was taken to the operating room for a laparotomy. Intraoperative findings included 4.5 liters of hemoperitoneum, a cervical stump pregnancy with well-formed fetus and intact gestational sac, and active bleeding from partially detached placental site. While the left ovary appeared normal, the right ovary and both fallopian tubes were absent.

The ectopic gestation was clamped at its base and resected from the cervical stump. Bleeding sites were ligated to ensure hemostasis. Our patient was deemed too unstable for trachelectomy. Her abdomen was irrigated with warm saline and the incision closed in layers. She was transferred to our intensive care unit (ICU) for aggressive volume resuscitation with 8 liters of normal saline and 5 units of blood, and a dopamine drip was initiated to assist with blood pressure control. After 2 hours, her blood pressure had stabilized and she was discharged 9 days later. On follow-up at fourth week post-operation, she was healing well and had hemoglobin of 11 g/dl.

## Discussion and conclusions

Pregnancy after hysterectomy can follow any type of hysterectomy (total or supracervical) and any approach (abdominal, laparoscopic, or vaginal), but the highest risk is with supracervical hysterectomies [3, 5]. Pregnancies after hysterectomy can take one of two forms: early and late presenting.

### Early presentation

Among 72 reported cases worldwide, 30 occurred because of unrecognized luteal phase pregnancies that were in transit to the endometrial cavity. These are considered early presenting post-hysterectomy pregnancies. The other possibility in this category is that sperm were present within the fallopian tube when the hysterectomy was performed.

### Late presentation

Late-presenting pregnancies develop as a result of a communication between the vagina and the peritoneal cavity. The location of these ectopic pregnancies after hysterectomy depends on the type of hysterectomy performed and the presence or absence of a residual cervix [3]. Of these cases, 50% have followed vaginal hysterectomy [2].

Our case was a late-presenting ectopic pregnancy following supracervical hysterectomy, probably from excess residual tissue. This is probably the third case of cervical stump pregnancy next to the two cases reported by McDaniel and Gullo in 1968 [6]. The interval of time between hysterectomy and ectopic pregnancy for our patient was 6 years, and this contributed to delay in seeking medical care and to late diagnosis. Such pregnancies have been reported from 2 months to 12 years after hysterectomy [1, 4]. Delay in diagnosis and treatment is the leading cause of complication and death from ectopic pregnancies [4]. In fact, our patient was close to death by the time the diagnosis was made.

### Prevention

Elective hysterectomies should be done in the pre-ovulatory phase of the menstrual cycle or after effective contraception to avoid early occurring post-hysterectomy ectopic pregnancies [2, 3]. In supracervical hysterectomy, removing as much tissue as possible will decrease the risk of ectopic pregnancy [5, 7]. In our case, our patient’s unstable condition precluded trachelectomy, and our main goal was to secure hemostasis and shorten anesthesia time. In 1921, McMillan and Dunn reported the case of an 18-year-old patient who experienced two pregnancies following hysterectomy [1]. The first pregnancy occurred 18 months after subtotal hysterectomy. The second pregnancy 17 months later resulted in the death of the patient from hemorrhage [1]. While unlikely, we understand that our patient may develop a second ectopic pregnancy in the future and are considering an elective trachelectomy to prevent this.

### Conclusion

While rare, post-hysterectomy ectopic pregnancy can occur and should be considered when a woman presents with abdominal pain and bleeding early or late after hysterectomy.

## Abbreviations

hCG: Human chorionic gonadotropin
ICU: Intensive care unit
